# Method of Tremor Levodopa Responsiveness Assessment and Its Correlation With Clinical Factors in Parkinson's Disease: Outcomes of the Acute Levodopa Challenge Test

**DOI:** 10.1155/padi/9923049

**Published:** 2025-05-20

**Authors:** Fangfei Li, Shaosong Xing, Yusha Cui, Lingyan Ma, Rui Yan, Genliang Liu, Tao Feng

**Affiliations:** ^1^Department of Neurology, Beijing Chaoyang Hospital, Capital Medical University, Beijing, China; ^2^Center for Movement Disorders, Department of Neurology, Beijing Tiantan Hospital, Capital Medical University, Beijing, China; ^3^Department of Emergency, Northern Medical Branch of PLA General Hospital, Beijing, China; ^4^China National Clinical Research Center for Neurological Diseases, Beijing, China

**Keywords:** acute levodopa challenge test, factor, levodopa responsiveness, Parkinson's disease, tremor, Unified Parkinson Disease Rating Scale

## Abstract

**Objectives:** Levodopa remains the most effective treatment for Parkinson's disease (PD); however, tremor reactions to dopaminergic medications show significant variance among patients with PD. This study aimed to assess the different methodologies employed to determine the dopamine responsiveness of tremors and their association with the clinical characteristics of PD.

**Methods:** Patients with PD and tremors were evaluated using the acute levodopa challenge test (LCT). Tremor levodopa responsiveness (LR) was calculated using the Unified Parkinson's Disease Rating Scale Part III (UPDRS-III) scores during OFF and ON periods. Tremor LR was calculated in two formats: absolute difference in tremor scores (OFF–ON), termed aLR, and percentage change in tremor scores, termed %LR and calculated as ([OFF–ON]/OFF100%). Independent variables were compared between the better tremor response to levodopa and poorer tremor response to levodopa groups based on the tremor change rate median score. Additionally, the effect of the tremor LR calculation method was correlated with clinical measures.

**Results:** This study enrolled 188 patients with PD who displayed tremors, of whom 98 (52%) showed better tremor response to levodopa. We identified a moderately negative correlation between tremor aLR and the rigidity-to-tremor score ratio (*r* = 0.40) during the OFF period, in addition to positive correlations between tremor aLR and the tremor score (*r* = 0.75), rest tremor score (*r* = 0.75), motor score (*r* = 0.42), postural and kinetic tremor score (*r* = 0.30), and tremor score-to-disease duration ratio (*r* = 0.30) of the UPDRS-III during OFF periods. The tremor %LR showed no significant relationship with any of the tested variables.

**Conclusions:** The aLR, rather than the %LR, is a more effective assessment method for evaluating the efficacy of levodopa for treating tremors in PD.

## 1. Introduction

Parkinson's disease (PD) is a progressive neurodegenerative disorder characterized by reduced dopamine levels in the nigrostriatal region of the brain [1]. Although dopaminergic drugs such as levodopa can effectively control bradykinesia and rigidity, their effect on tremors is less predictable, with significant variation between patients, although the underlying reasons are not well understood [2, 3]. Understanding levodopa responsiveness (LR) in tremors is critical to achieve a mechanistic understanding and facilitate rational drug use.

The acute levodopa challenge test (LCT) is widely used in both the clinical and research settings to measure motor improvement after levodopa administration. This improvement, reflected in the Unified Parkinson's Disease Rating Scale Part III (UPDRS-III) scores during the test, is termed as the LR. The decrease in the score from the OFF state to the ON state reflects the patient's response to levodopa. LR can be quantified either as the absolute score difference between the OFF and ON states (absolute LR or aLR) [4] or as a percentage change (%LR) in scores [5]. However, there is currently no clear consensus on the optimal method for calculating the LR of tremors in patients with PD. In our study, we assessed the tremor aLR and tremor %LR, and stratified patients with PD with tremors into the better tremor response to levodopa and poorer tremor response to levodopa groups according to the tremor change rate median score [6, 7]. Highlighting a better method for evaluating dopamine responsiveness in patients with PD with tremors could help clinicians to make informed treatment decisions and guide researchers in selecting appropriate methods.

Previous research on PD has shown inconsistent results regarding the association between clinical characteristics and LR. Some studies have identified a significant negative association between age and drug response, with gait and bradykinesia being more affected by age than by rest tremor [4]. In contrast, one study found no association between disease duration and LR, instead revealing a notable inverse relationship between age and LR, except in tremor-dominant PD [5]. In another study, Zach et al. found that patients with dopamine-responsive PD with tremors had greater disease severity, longer disease duration, and a higher incidence of dyskinesia [8]. Comparisons between the dopamine-responsive and dopamine-resistant groups have yielded mixed results regarding differences in sex, levodopa equivalent daily dose (LEDD), disease duration and Hoehn–Yahr stage (H–Y stage) [9, 10]. Due to the heterogeneity in the results from these studies, in the present study, we compared the differences in demographic, clinical, and treatment data between patients with PD with better responsiveness of PD tremor to LR and poorer responsiveness of PD tremor to levodopa. Additionally, we examined how tremor LR was related to factors such as age, disease duration, OFF-motor scores, and various clinical scales.

Given the lack of consensus on the optimal method for calculating tremor LR and the variability in tremor response to dopaminergic treatment, the management of patients with PD and severe tremors can be challenging for clinicians. As such, this study aimed to evaluate the factors associated with the efficacy of levodopa treatment in patients with PD and tremors, in order to analyze the method used to calculate tremor LR, identify the related clinical factors associated with dopamine responsiveness, provide evidence supporting individualized drug use, and investigate whether the heterogeneity of the dopamine response in PD may involve different mechanisms.

## 2. Methods

### 2.1. Patients

A cohort of 188 patients with PD was enrolled from the Movement Disorder Center, Department of Neurology, Beijing Tiantan Hospital and Capital Medical University (Beijing, China). This study was approved by the Institutional Review Board of Beijing Tiantan Hospital (KY2024-037-02). The inclusion criteria were as follows: (1) those aged between 45 and 85 years; (2) those confirmed diagnosis of PD based on the 2015 MDS diagnostic criteria [11, 12]; and (3) those who had at least one UPDRS-III item: postural tremor, intention tremor, and resting hand tremor scored ≥ 1. The exclusion criteria for participation in this study were as follows: (1) those who had history of brain surgery, such as deep brain stimulation, (2) those with the presence of brain tumors or other neurological disorders, (3) those with any severe physical disabilities that could interfere with mobility assessment; and (4) those with any cognitive impairments or psychological conditions that could prevent the completion of all required procedures. Written informed consent was obtained from all participants.

### 2.2. Assessments

Patient demographics such as sex, age at onset, disease duration, LEDD, orthostatic hypotension (OH), H–Y stage, and UPDRS-III scores were assessed and recorded by movement disorder specialists.

OH manifests as a sustained fall in systolic blood pressure of 20 mmHg or more, or a diastolic blood pressure of 10 mmHg, within the first 3 minutes of standing up from a lying position. Symptoms may include dizziness, fainting, and lightheadedness, but this condition may also present with no symptoms [13].

In this study, the UPDRS-III scores and subsymptoms of tremor, bradykinesia, rigidity, and postural instability were recorded during the OFF and ON phases of acute LCT. In addition, seven score components were identified according to the Movement Disorder Society criteria, as follows: midline function, rest tremor, rigidity, upper limb bradykinesia of the more affected side (MAS) and the less affected side (LAS), postural and kinetic tremor, and lower limb bradykinesia, according to the Movement Disorder Society [14]. In addition, we calculated the disease progression index as the ratio of the UPDRS-III scores to disease duration. Patients were categorized into better tremor response to levodopa and poorer tremor response to levodopa groups, based on the tremor change rate median score [6]. Those with a tremor change rate score > 0.45 were classified as better tremor response to the levodopa group, while those with a score ≤ 0.45 were classified as poorer tremor response to the levodopa group.

### 2.3. LCT

The participants underwent LCT according to the CAPSIT PD guidelines [15]. LED was recorded by movement disorder specialists. The test was conducted in the morning, 36 h after withdrawal from dopamine agonists with an overnight withdrawal from levodopa and other anti-Parkinsonian drugs, to ensure a sufficient washout period. The dose for LCT was set at a dose equivalent to 150% of the usual morning LED. An immediate-release levodopa formulation containing 25% benserazide was prepared. The UPDRS-III was administered initially in the OFF condition and then repeated at 30-min intervals for five hours after administration. The analysis focused on the highest LR recorded for each patient.

ON and OFF tremor scores were evaluated for each participant, and levodopa tremor responsiveness was calculated as the absolute difference ([OFF–ON]; absolute LR or aLR) and percentage change ([OFF–ON]/OFF100%; %LR).

### 2.4. Statistical Analysis

Data are presented as means with standard deviations, medians with interquartile ranges, or numbers with percentages. Initial analyses using two-sample *t* tests, Pearson's chi-square tests, or nonparametric tests, as appropriate, were applied to distinguish the better response of PD tremor to levodopa and poorer response of PD tremor to levodopa groups. Multivariate linear regression analysis was performed using tremor LR as the dependent variable, while independent variables were identified as significant using Pearson's correlation analysis. Correlation strengths were defined as follows: ≥ 0.8 indicated a very strong correlation; 0.6–0.8, moderate; 0.3–0.5, fair; and < 0.3, poor [16]. Statistical analyses were performed using SPSS 23.0, with statistical significance set at *p* < 0.05.

## 3. Results

### 3.1. Patients

A cohort of 188 patients was included, for whom demographic and disease-specific data were collected during the OFF phase, as shown in Table 1.

### 3.2. Difference of Tremor LR Between Better Tremor Response to Levodopa and Poorer Tremor Response to Levodopa

The demographic and clinical variables were compared between the better tremor response to levodopa (*N* = 98) and poorer tremor response to levodopa groups (*N* = 90) (Table 2). Comparative analyses revealed higher scores for tremors and resting tremors in the group with better responsiveness of PD tremor to levodopa (*p*=0.008). No significant differences were found in age, age of onset, disease duration, sex, LED, H–Y stage, OH, postural instability gait disorder score, hypokinesia score, rigidity score, upper- or lower-limb bradykinesia score, postural and kinetic tremor score, or motor score (*p* > 0.05). There were also no differences in the ratios of tremor, postural instability, gait disorder, hypokinesia, or rigidity scores to disease duration between the two groups in the OFF state (*p* > 0.05).

### 3.3. Association Involving Variables and Tremor LR

Correlation analysis was performed to examine the association between the LR of tremors and various demographic and disease factors, including age, onset age, disease duration, sex, LED, H–Y stage, and UPDRS-III score. As shown in Table 3, our analysis identified positive correlations between tremor aLR and OFF tremor scores (*r* = 0.75, *p* < 0.001), OFF rest tremor score (*r* = 0.75, *p* < 0.001), OFF motor score (*r* = 0.42, *p* < 0.001), OFF postural and kinetic tremor scores (*r* = 0.30, *p* < 0.001), and the ratio of OFF tremor score to disease duration ratio (*r* = 0.30, *p* < 0.001). Overall, there was a negative correlation between tremor aLR and the OFF rigidity-to-tremor score ratio (*r* = 0.40, *p* < 0.001). Poor correlations were further observed between tremor aLR and OFF lower-limb bradykinesia score, OFF midline function score, disease duration, OFF bradykinesia score, OFF postural instability score, OFF rigidity score, OFF bradykinesia MAS/LAS upper-limb score, H–Y stage, and LED (*r* < 0.3, *p* < 0.05). In addition, no clear associations were found between tremor aLR and variables such as age, sex, age at onset, and OH level (*p* > 0.05). In addition, the %LR was not significantly correlated with any of the assessed factors (*p* > 0.05, Table 4).

## 4. Discussion

This study currently used tremor LR calculation methods and investigated the factors influencing dopamine responsiveness. This comparison identified three main findings: First, the aLR for tremor proved to be a more effective index for documenting tremor responses to levodopa in patients with PD than the %LR, as it correlated significantly with clinical factors. Second, patients with the better responsiveness of PD tremor to levodopa had higher tremor and resting tremor scores on the UPDRS-III. Third, a correlation was observed between tremor aLR and motor symptoms, including tremor symptoms, the ratio of rigidity-to-tremor score, and the tremor progression index as a ratio of tremor score-to-disease duration. These findings indicate that understanding the LR of tremors may provide evidence for rational drug use.

Achieving consistency in acute motor response to levodopa, traditionally measured as the %LR after a minimum of 12 h without medication, is challenging. Different patients show different actual changes with the same percentage of change. For example, a patient in the early stages of the disease (Patient A) showed a decrease in the UPDRS-III tremor score from 10 to 5, representing a 50% LR, but only a 5-point absolute reduction. Conversely, a patient with a more advanced stage (Patient B) showed the same 50% LR, progressing from a score of 20 to 10, with an absolute change of 10 points. As such, reporting tremor %LR alone may be misleading. In this study, tremor %LR showed no significant correlation with any of the demographic or clinical variables. In contrast, tremor aLR was significantly associated with tremor and rest tremor scores and showed fair correlations with motor score, rigidity-to-tremor score ratio, postural and kinetic tremor score, and tremor score-to-disease duration ratio. However, tremor aLR did not correlate with age, disease duration, H–Y stage, or LED. Accordingly, our results suggest that the aLR provides a more accurate reflection of PD tremor characteristics than the %LR and is therefore a more sensitive indicator for assessing tremor response levels.

Previous studies in PD have shown inconsistencies in the results of correlations between the LR of tremors and age, sex, and disease duration [4, 8, 9]. For example, Zach et al. found that dopamine-responsive PD tremors are associated with greater disease severity [8]. Another study comparing dopamine-responsive and nonresponsive PD tremor groups found that the scores for rigidity, akinesia, postural instability, UPDRS III total score, and H–Y stage were significantly higher in the dopamine-responsive group [10]. A recent prospective study found all three cardinal symptoms similarly responsive [17]. In our study, we found that tremor scores were different between two groups, whereas other motor symptom scores such as rigidity and bradykinesia were not different between better tremor response to levodopa and poorer response to levodopa groups. The inconsistency in these findings suggests that they may be related to different grouping methods and different enrolled population. Our results confirmed that more advanced PD cases are likely to have a significant levodopa response, particularly those with more severe tremors and rest tremors, but not with other major motor symptoms, age, sex, or disease duration. This is also consistent with Swinnen et al.'s conclusion that tremor in advanced PD may be more sensitive than other major motor features [18].

Numerous mechanisms may be involved in the maintenance of response amplitudes as degenerative processes progress. One such mechanism involves the enhancement of postsynaptic striatal *D*_2_ receptors, as highlighted by Aygun et al. [5]. In conditions such as multiple system atrophy, particularly the Parkinsonian subtype, both the striatum and the substantia nigra pars compacta (SNc) undergo significant degeneration [19]. Imaging results have showed a significant reduction in both pre- and postsynaptic striatal *D*_2_ receptor activity in patients with MSA. Such degeneration in MSA, the Parkinsonian subtype, is correlated with a reduced response to levodopa treatment [20, 21]. Conversely, the striatum in patients with PD shows less degeneration, allowing for an increase in *D*_2_ receptors, as confirmed by studies using positron emission tomography [22]. This relatively preserved striatal state in PD may help to maintain robust tremor amplitude responses to levodopa, as the SNc terminals deteriorate, exacerbating tremor symptoms and disease progression. However, *D*_2_ receptor upregulation in response to dopaminergic degeneration was relevant in the untreated stage of PD but probably irrelevant after years of treatment [23]. This suggested that other neurotransmitter systems and brain areas may indeed play a role in PD tremor. Future studies should stratify PD patients by treatment history to investigate dopaminergic responsiveness of tremor.

Our observations showed a negative correlation between the ratio of rigidity-to-tremor score and tremor aLR, whereas we observed a positive correlation between the tremor progression index and tremor aLR in PD. Sung et al. found that patients with PD had a more pronounced response to dopaminergic treatments for resting tremors when accompanied by moderate bradykinesia and rigidity rather than in isolation [10]. This finding is consistent with our findings that the dopamine-responsive group shows higher scores for tremors and other symptoms. However, at the same time, the severity of tremors and rigidity were not proportional. As described by Lees et al. [24], the OFF score is a reliable indicator of SNc degeneration. As a result, individuals with an elevated aLR may experience an intensification of tremor symptoms, suggesting possible progression to a more advanced disease stage due to the involvement of Lewy body pathology in additional critical regions.

Previous studies, such as those using ^18^F-dopa PET scans, have shown that reductions in ^18^F-fluorodopa uptake in the striatum are associated with the severity of bradykinesia and rigidity, but not necessarily with the severity of tremors [25]. The recent research has explored the relationship between resting tremors in PD and deficits in nondopaminergic neurotransmitters, particularly serotonin [26, 27]. Other studies have further analyzed tremor-related brain activity during medication sessions, showing that patients with dopamine-resistant tremor have increased activity in nondopaminergic areas, such as the cerebellum, while the dopamine-responsive group has increased activity in the thalamus and secondary somatosensory cortex [9]. There is also evidence to suggest that dopaminergic dysfunction may cause all or part of the remaining tremor in some patients with PD, indicating a highly variable response to dopaminergic treatments. Overall, our study provides evidence that the heterogeneity of the dopamine response in PD tremors may share different mechanisms. This suggests that the UPDRS-III scores may be more responsive to symptoms in dopaminergic areas than in nondopaminergic areas. Analysis of the MRI and electromyogram characteristics is required for further studies. This variability may require individualized treatment strategies based on underlying pathophysiological processes.

This study had several limitations. First, the assessment of motor and nonmotor symptoms was subjective and may have differed from that of movement disorder specialists. Second, this study did not analyze different types of tremors based on LR, and future research should expand the sample size to better understand the prognosis of different tremor subtypes. Third, although we fully investigated clinical and demographic characteristics, other factors, including MRI and electromyography characteristics, may require further investigation. Fourth, due to the single-center nature of the study and regional prescribing patterns, future multicenter studies including diverse ethnic populations would help validate these observations. Finally, this was not a longitudinal study, and we did not further differentiate the effect of the long-duration response and short-duration response of untreated PD or long-term treated PD; as such, future studies involving intensive follow-ups and expansion of the scope of the investigation to further subgroup analyses should be conducted to obtain more accurate results. Understanding these dynamics may significantly improve our understanding of PD.

## 5. Conclusion

Overall, despite the widespread use of %LR in clinical and research protocols, the results of the present study support tremor aLR as a more accurate index for assessing tremor response in PD than in %LR because of its significant association with many clinical factors.

## Figures and Tables

**Table 1:** Demographic and disease information (*N* = 188).

Variables	Mean (SD)	Range
Age	66.28 (10.20)	30–90
Sex; male: female	95M: 93F	—
Disease duration	8.57 (5.57)	0.67–57.67
Onset age	56.86 (10.42)	40–82
LEDD^a^	308.43 (196.41)	37.50–875
LED^b^	165.08 (48.17)	50–294
OFF; UPDRS-III	42.08 (18.22)	5–98
ON; UPDRS-III	27.02 (14.44)	4–84
Tremor %LR	0.44 (0.37)	−1–1
Tremor aLR	3.64 (3.69)	−4–19
H–Y stage	2.82 (0.89)	1–5

*Note:* UPDRS-III, Unified Parkinson Disease Rating Scale Part III; %LR, percentage change levodopa response.

Abbreviations: aLR, absolute levodopa response; H–Y stage, Hoehn–Yahr stage; LED, levodopa equivalent dose; LEDD, levodopa equivalent daily dose.

^a^Note that LEDD refers to the values of medicated patients only (*N* = 140).

^b^Note that LED refers to the levodopa equivalent dose of anti-Parkinsonian medication used in levodopa challenge test in the morning.

**Table 2:** Demographic and clinical variables between two groups.

Variables	Poorer tremor response to levodopa group (*N* = 90)	Better tremor response to levodopa group (*N* = 98)	*p*
Age	68.00 (13.75)	69.00 (13)	0.465
Sex (female%)	43.00 (47.80)	50.00 (51.00)	0.766
Onset age	58.00 (10.75)	58.50 (13.00)	0.969
Disease duration	7.08 (5.46)	8.42 (4.10)	0.0549
H–Y stage	3.00 (1.00)	3.00 (0.50)	0.334
LED^a^	150.00 (50.00)	150.00 (50.00)	0.474
OH (%)	24.00 (26.70)	38.00 (38.80)	0.108
OFF tremor score^∗∗^	5.00 (6.00)	8.00 (6.50)	0.008
OFF rest tremor score^∗∗^	3.50 (5.00)	6.00 (6.00)	0.008
OFF postural instability score	5.00 (5.00)	6.00 (5.00)	0.232
OFF hypokinesia score	15.00 (11.00)	15.50 (14.00)	0.819
OFF rigidity score	8.00 (4.00)	8.00 (7.00)	0.574
OFF midline function score	9.00 (6.50)	11.00 (6.00)	0.138
OFF bradykinesia MAS upper extremity score	5.00 (4.00)	5.00 (4.00)	0.852
OFF bradykinesia LAS upper extremity score	3.00 (4.00)	3.00 (4.00)	0.895
OFF postural and kinetic tremors score	2.00 (3.00)	2.00 (3.00)	0.814
OFF lower limb bradykinesia score	5.50 (5.00)	5.00 (5.50)	0.476
OFF motor score	38.50 (22.50)	38.50 (24.40)	0.261
OFF rigidity-to-tremor score ratio	1.33 (1.92)	1.09 (0.96)	0.080
OFF tremor score-to-disease duration ratio	0.83 (0.93)	0.93 (0.92)	0.254
OFF motor score-to-disease duration ratio	5.40 (4.48)	4.99 (3.54)	0.426
OFF postural instability score-to-disease duration ratio	0.84 (0.88)	0.75 (0.55)	0.538
OFF rigidity score-to-disease duration ratio	1.08 (1.08)	1.00 (0.86)	0.248
OFF hypokinesia score-to-disease duration ratio	1.98 (2.60)	1.98 (1.87)	0.260

Abbreviations: H–Y stage, Hoehn–Yahr stage; LAS, less affected sides; LED, levodopa equivalent dose; MAS, more affected sides.

^a^Note that LED refers to the levodopa equivalent dose of anti-Parkinsonian medication used in levodopa challenge test in the morning.

^∗∗^indicates *p* < 0.01.

**Table 3:** The correlations between the variables and the tremor aLR.

Variables	*r*	Tremor aLR *p*
OFF tremor score	0.75^∗∗∗^	< 0.001
OFF rest tremor score	0.75^∗∗∗^	< 0.001
OFF motor score	0.42^∗∗∗^	< 0.001
OFF rigidity-to-tremor score ratio	−0.40^∗∗∗^	< 0.001
OFF postural and kinetic tremor score	0.30^∗∗∗^	< 0.001
OFF tremor score-to-disease duration ratio	0.30^∗∗∗^	< 0.001
OFF lower limb bradykinesia score	0.26^∗∗∗^	< 0.001
OFF midline function score	0.23^∗∗^	0.001
Disease duration	0.21^∗∗^	0.003
OFF bradykinesia score	0.21^∗∗^	0.003
OFF postural instability score	0.21^∗∗^	0.003
OFF rigidity score	0.20^∗∗^	0.004
OFF bradykinesia MAS upper extremity score	0.20^∗∗^	0.004
H–Y stage	0.20^∗∗^	0.005
OFF bradykinesia LAS upper extremity score	0.16^∗^	0.030
LED^a^	0.15^∗^	0.040

Abbreviations: aLR, absolute levodopa response; H–Y stage, Hoehn–Yahr stage; LAS, less affected sides; LED, levodopa equivalent dose; MAS, more affected sides.

^a^Note that LED refers to the levodopa equivalent dose of anti-Parkinsonian medication used in levodopa challenge test in the morning.

^∗^indicates *p* < 0.05.

^∗∗^indicates *p* < 0.01.

^∗∗∗^indicates *p* < 0.001.

**Table 4:** The correlations between the variables and the tremor %LR.

Variables	*r*	Tremor %LR *p*
OFF rest tremor score	0.31^∗∗∗^	< 0.001
OFF tremor score	0.25^∗∗∗^	< 0.001
OFF rigidity-to-tremor score ratio	−0.20^∗∗^	0.004
Disease duration	0.14^∗^	0.045

*Note:* %LR, percentage change levodopa response.

^∗^indicates *p* < 0.05.

^∗∗^indicates *p* < 0.01.

^∗∗∗^indicates *p* < 0.001.

## Data Availability

Data are available upon reasonable request to the corresponding author.
